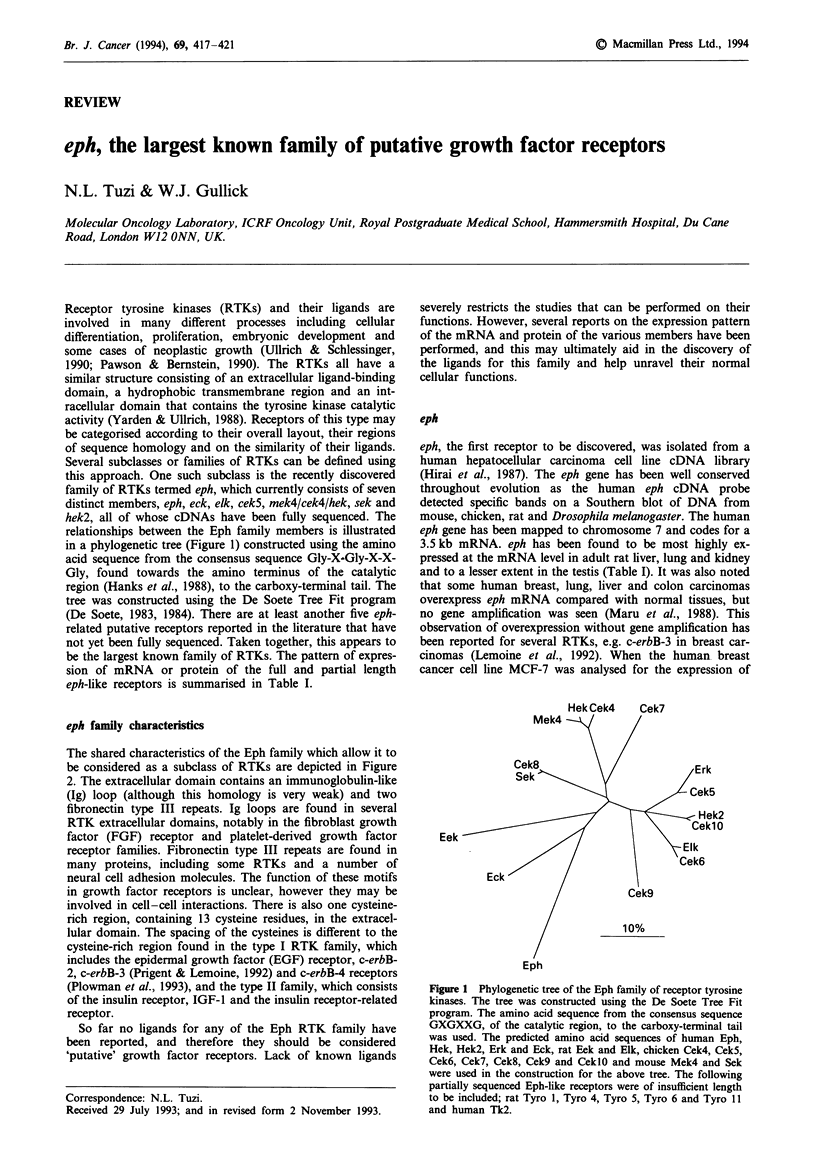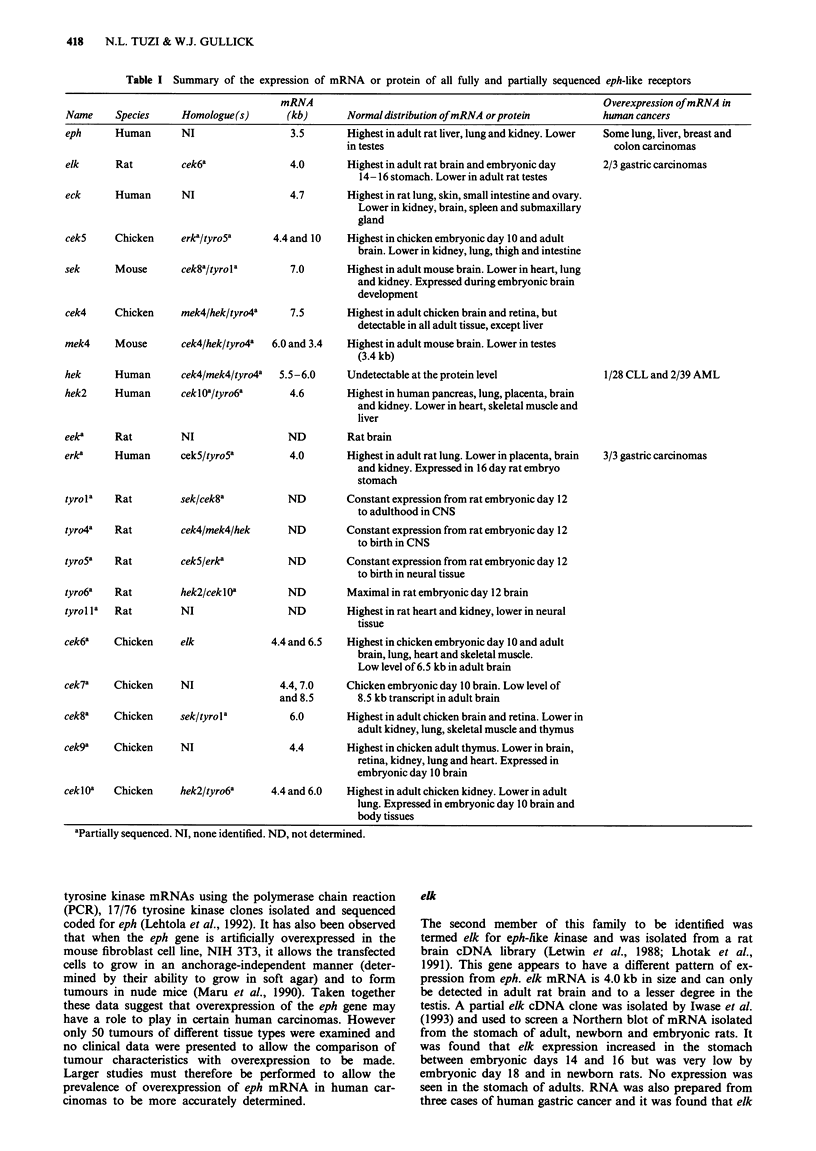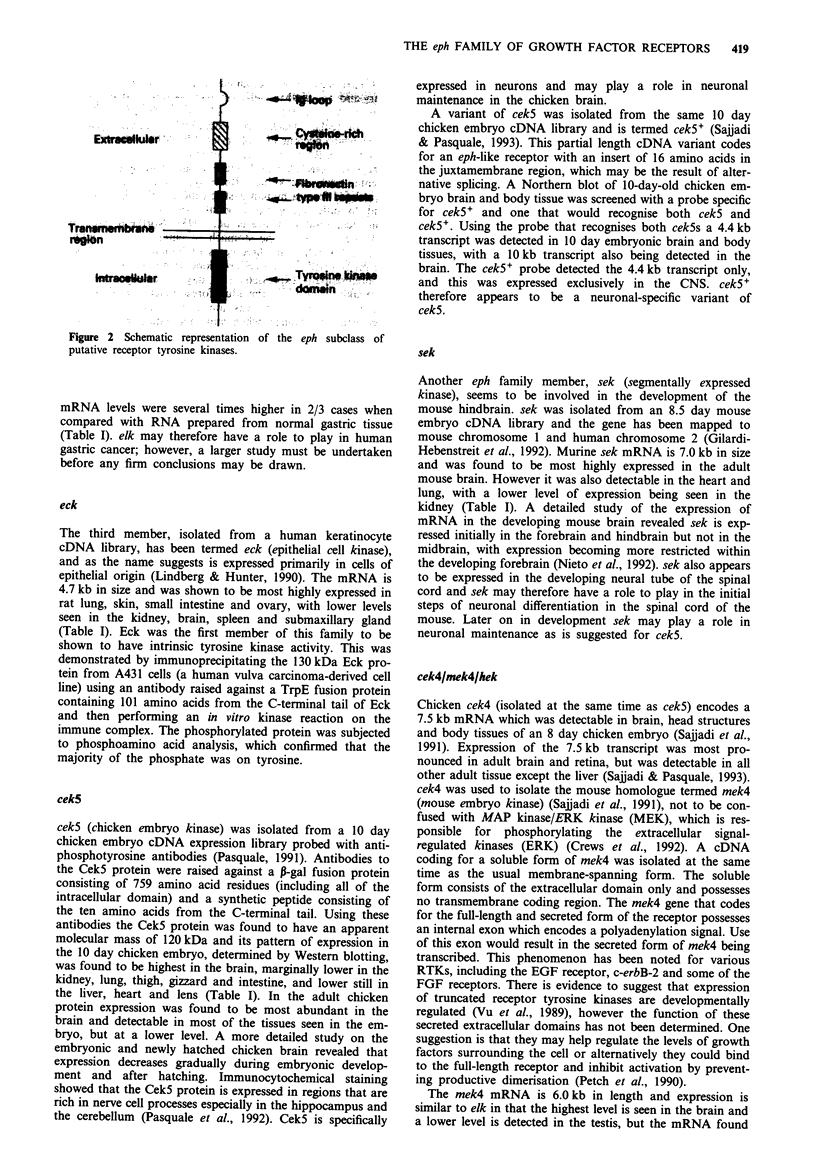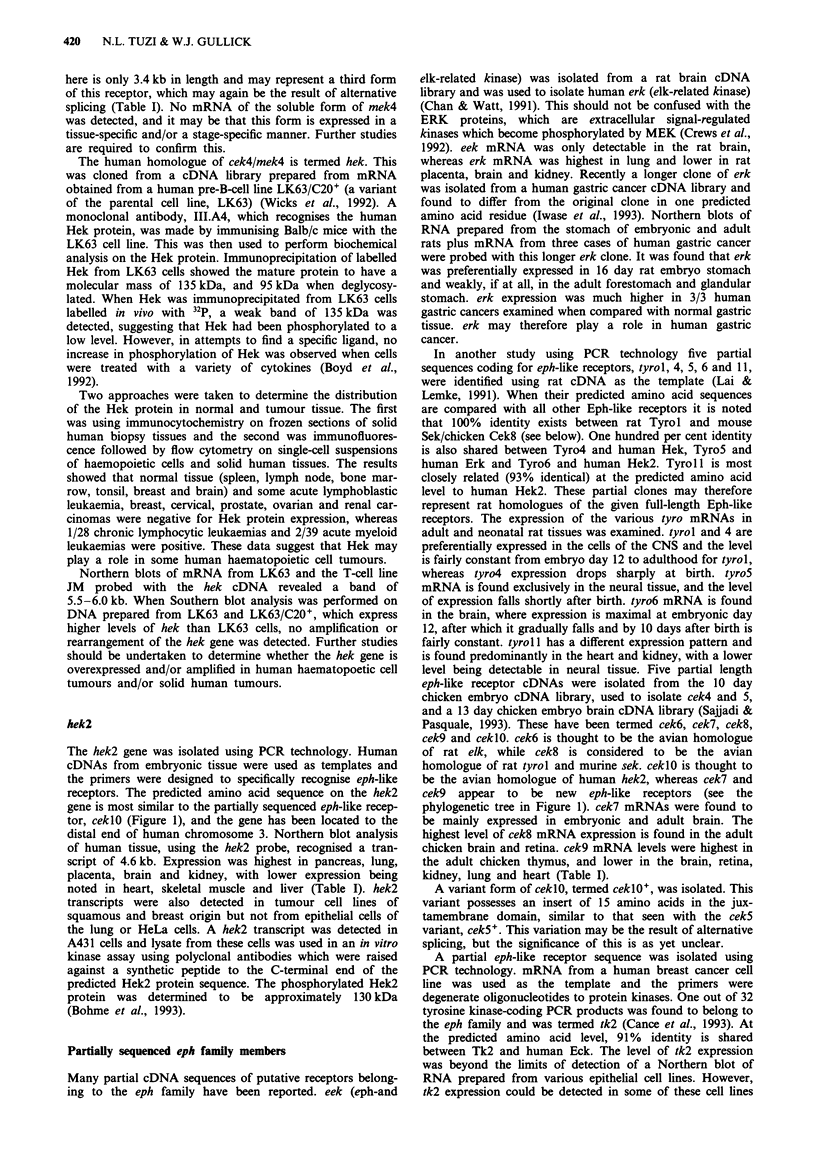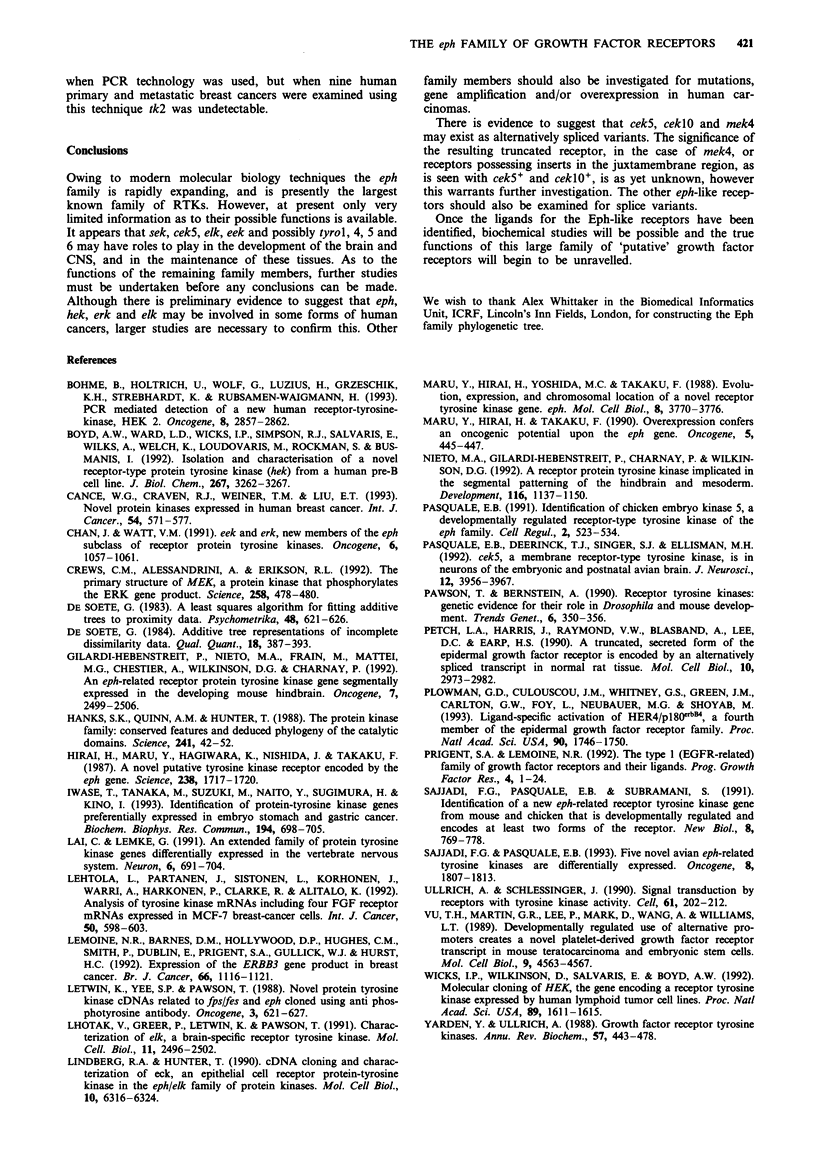# eph, the largest known family of putative growth factor receptors.

**DOI:** 10.1038/bjc.1994.77

**Published:** 1994-03

**Authors:** N. L. Tuzi, W. J. Gullick

**Affiliations:** Molecular Oncology Laboratory, Royal Postgraduate Medical School, Hammersmith Hospital, London, UK.


Br. J. Cancer (1994), 69, 417-421 © Macmillan Press Ltd., 1994

REVIEW

### *eph*, the largest known family of putative growth factor receptors

N.L. Tuzi & W.J. Gullick

*Molecular Oncology Laboratory, ICRF Oncology Unit, Royal Postgraduate Medical School, Hammersmith Hospital, Du Cane Road, London W12 0NN, UK.*

Receptor tyrosine kinases (RTKs) and their ligands are involved in many different processes including cellular differentiation, proliferation, embryonic development and some cases of neoplastic growth (Ullrich & Schlessinger, 1990; Pawson & Bernstein, 1990). The RTKs all have a similar structure consisting of an extracellular ligand-binding domain, a hydrophobic transmembrane region and an intracellular domain that contains the tyrosine kinase catalytic activity (Yarden & Ullrich, 1988). Receptors of this type may be categorised according to their overall layout, their regions of sequence homology and on the similarity of their ligands. Several subclasses or families of RTKs can be defined using this approach. One such subclass is the recently discovered family of RTKs termed *eph*, which currently consists of seven distinct members, *eph*, *eck*, *elk*, *cek*5, *mek*4/*cek*4/*hek*, *sek* and *hek*2, all of whose cDNAs have been fully sequenced. The relationships between the Eph family members is illustrated in a phylogenetic tree (Figure 1) constructed using the amino acid sequence from the consensus sequence Gly-X-Gly-X-X-Gly, found towards the amino terminus of the catalytic region (Hanks *et al.*, 1988), to the carboxy-terminal tail. The tree was constructed using the De Soete Tree Fit program (De Soete, 1983, 1984). There are at least another five *eph*-related putative receptors reported in the literature that have not yet been fully sequenced. Taken together, this appears to be the largest known family of RTKs. The pattern of expression of mRNA or protein of the full and partial length *eph*-like receptors is summarised in Table I.

### *eph* family characteristics

The shared characteristics of the Eph family which allow it to be considered as a subclass of RTKs are depicted in Figure 2. The extracellular domain contains an immunoglobulin-like (Ig) loop (although this homology is very weak) and two fibronectin type III repeats. Ig loops are found in several RTK extracellular domains, notably in the fibroblast growth factor (FGF) receptor and platelet-derived growth factor receptor families. Fibronectin type III repeats are found in many proteins, including some RTKs and a number of neural cell adhesion molecules. The function of these motifs in growth factor receptors is unclear, however they may be involved in cell-cell interactions. There is also one cysteine-rich region, containing 13 cysteine residues, in the extracellular domain. The spacing of the cysteines is different to the cysteine-rich region found in the type I RTK family, which includes the epidermal growth factor (EGF) receptor, c-*erb*B-2, c-*erb*B-3 (Prigent & Lemoine, 1992) and c-*erb*B-4 receptors (Plowman *et al.*, 1993), and the type II family, which consists of the insulin receptor, IGF-1 and the insulin receptor-related receptor.

So far no ligands for any of the Eph RTK family have been reported, and therefore they should be considered 'putative' growth factor receptors. Lack of known ligands severely restricts the studies that can be performed on their functions. However, several reports on the expression pattern of the mRNA and protein of the various members have been performed, and this may ultimately aid in the discovery of the ligands for this family and help unravel their normal cellular functions.

### *eph*

*eph*, the first receptor to be discovered, was isolated from a human hepatocellular carcinoma cell line cDNA library (Hirai *et al.*, 1987). The *eph* gene has been well conserved throughout evolution as the human *eph* cDNA probe detected specific bands on a Southern blot of DNA from mouse, chicken, rat and *Drosophila melanogaster*. The human *eph* gene has been mapped to chromosome 7 and codes for a 3.5 kb mRNA. *eph* has been found to be most highly expressed at the mRNA level in adult rat liver, lung and kidney and to a lesser extent in the testis (Table I). It was also noted that some human breast, lung, liver and colon carcinomas overexpress *eph* mRNA compared with normal tissues, but no gene amplification was seen (Maru *et al.*, 1988). This observation of overexpression without gene amplification has been reported for several RTKs, e.g. c-*erb*B-3 in breast carcinomas (Lemoine *et al.*, 1992). When the human breast cancer cell line MCF-7 was analysed for the expression of

**Figure 1** Phylogenetic tree of the Eph family of receptor tyrosine kinases. The tree was constructed using the De Soete Tree Fit program. The amino acid sequence from the consensus sequence GXGXXG, of the catalytic region, to the carboxy-terminal tail was used. The predicted amino acid sequences of human Eph, Hek, Hek2, Erk and Eck, rat Eek and Elk, chicken Cek4, Cek5, Cek6, Cek7, Cek8, Cek9 and Cek10 and mouse Mek4 and Sek were used in the construction for the above tree. The following partially sequenced Eph-like receptors were of insufficient length to be included; rat Tyro 1, Tyro 4, Tyro 5, Tyro 6 and Tyro 11 and human Tk2.

Correspondence: N.L. Tuzi.
Received 29 July 1993; and in revised form 2 November 1993.

**Table I** Summary of the expression of mRNA or protein of all fully and partially sequenced *eph*-like receptors

| *Name* | *Species* | *Homologue(s)* | *mRNA (kb)* | *Normal distribution of mRNA or protein* | *Overexpression of mRNA in human cancers* |
|---|---|---|---|---|---|
| *eph* | Human | NI | 3.5 | Highest in adult rat liver, lung and kidney. Lower in testes | Some lung, liver, breast and colon carcinomas |
| *elk* | Rat | *cek6*[a] | 4.0 | Highest in adult rat brain and embryonic day 14–16 stomach. Lower in adult rat testes | 2/3 gastric carcinomas |
| *eck* | Human | NI | 4.7 | Highest in rat lung, skin, small intestine and ovary. Lower in kidney, brain, spleen and submaxillary gland | |
| *cek5* | Chicken | *erk*[a]/*tyro5*[a] | 4.4 and 10 | Highest in chicken embryonic day 10 and adult brain. Lower in kidney, lung, thigh and intestine | |
| *sek* | Mouse | *cek8*[a]/*tyro1*[a] | 7.0 | Highest in adult mouse brain. Lower in heart, lung and kidney. Expressed during embryonic brain development | |
| *cek4* | Chicken | *mek4/hek/tyro4*[a] | 7.5 | Highest in adult chicken brain and retina, but detectable in all adult tissue, except liver | |
| *mek4* | Mouse | *cek4/hek/tyro4*[a] | 6.0 and 3.4 | Highest in adult mouse brain. Lower in testes (3.4 kb) | |
| *hek* | Human | *cek4/mek4/tyro4*[a] | 5.5–6.0 | Undetectable at the protein level | 1/28 CLL and 2/39 AML |
| *hek2* | Human | *cek10*[a]/*tyro6*[a] | 4.6 | Highest in human pancreas, lung, placenta, brain and kidney. Lower in heart, skeletal muscle and liver | |
| *eek*[a] | Rat | NI | ND | Rat brain | |
| *erk*[a] | Human | cek5/*tyro5*[a] | 4.0 | Highest in adult rat lung. Lower in placenta, brain and kidney. Expressed in 16 day rat embryo stomach | 3/3 gastric carcinomas |
| *tyro1*[a] | Rat | *sek/cek8*[a] | ND | Constant expression from rat embryonic day 12 to adulthood in CNS | |
| *tyro4*[a] | Rat | *cek4/mek4/hek* | ND | Constant expression from rat embryonic day 12 to birth in CNS | |
| *tyro5*[a] | Rat | *cek5/erk*[a] | ND | Constant expression from rat embryonic day 12 to birth in neural tissue | |
| *tyro6*[a] | Rat | *hek2/cek10*[a] | ND | Maximal in rat embryonic day 12 brain | |
| *tyro11*[a] | Rat | NI | ND | Highest in rat heart and kidney, lower in neural tissue | |
| *cek6*[a] | Chicken | *elk* | 4.4 and 6.5 | Highest in chicken embryonic day 10 and adult brain, lung, heart and skeletal muscle. Low level of 6.5 kb in adult brain | |
| *cek7*[a] | Chicken | NI | 4.4, 7.0 and 8.5 | Chicken embryonic day 10 brain. Low level of 8.5 kb transcript in adult brain | |
| *cek8*[a] | Chicken | *sek/tyro1*[a] | 6.0 | Highest in adult chicken brain and retina. Lower in adult kidney, lung, skeletal muscle and thymus | |
| *cek9*[a] | Chicken | NI | 4.4 | Highest in chicken adult thymus. Lower in brain, retina, kidney, lung and heart. Expressed in embryonic day 10 brain | |
| *cek10*[a] | Chicken | *hek2/tyro6*[a] | 4.4 and 6.0 | Highest in adult chicken kidney. Lower in adult lung. Expressed in embryonic day 10 brain and body tissues | |

[a]Partially sequenced. NI, none identified. ND, not determined.

tyrosine kinase mRNAs using the polymerase chain reaction (PCR), 17/76 tyrosine kinase clones isolated and sequenced coded for *eph* (Lehtola *et al.*, 1992). It has also been observed that when the *eph* gene is artificially overexpressed in the mouse fibroblast cell line, NIH 3T3, it allows the transfected cells to grow in an anchorage-independent manner (determined by their ability to grow in soft agar) and to form tumours in nude mice (Maru *et al.*, 1990). Taken together these data suggest that overexpression of the *eph* gene may have a role to play in certain human carcinomas. However only 50 tumours of different tissue types were examined and no clinical data were presented to allow the comparison of tumour characteristics with overexpression to be made. Larger studies must therefore be performed to allow the prevalence of overexpression of *eph* mRNA in human carcinomas to be more accurately determined.

### *elk*

The second member of this family to be identified was termed *elk* for *e*ph-*l*ike *k*inase and was isolated from a rat brain cDNA library (Letwin *et al.*, 1988; Lhotak *et al.*, 1991). This gene appears to have a different pattern of expression from *eph*. *elk* mRNA is 4.0 kb in size and can only be detected in adult rat brain and to a lesser degree in the testis. A partial *elk* cDNA clone was isolated by Iwase *et al.* (1993) and used to screen a Northern blot of mRNA isolated from the stomach of adult, newborn and embryonic rats. It was found that *elk* expression increased in the stomach between embryonic days 14 and 16 but was very low by embryonic day 18 and in newborn rats. No expression was seen in the stomach of adults. RNA was also prepared from three cases of human gastric cancer and it was found that *elk*

**Figure 2** Schematic representation of the *eph* subclass of putative receptor tyrosine kinases.

mRNA levels were several times higher in 2/3 cases when compared with RNA prepared from normal gastric tissue (Table I). *elk* may therefore have a role to play in human gastric cancer; however, a larger study must be undertaken before any firm conclusions may be drawn.

### *eck*

The third member, isolated from a human keratinocyte cDNA library, has been termed *eck* (*e*pithelial *c*ell *k*inase), and as the name suggests is expressed primarily in cells of epithelial origin (Lindberg & Hunter, 1990). The mRNA is 4.7 kb in size and was shown to be most highly expressed in rat lung, skin, small intestine and ovary, with lower levels seen in the kidney, brain, spleen and submaxillary gland (Table I). Eck was the first member of this family to be shown to have intrinsic tyrosine kinase activity. This was demonstrated by immunoprecipitating the 130 kDa Eck protein from A431 cells (a human vulva carcinoma-derived cell line) using an antibody raised against a TrpE fusion protein containing 101 amino acids from the C-terminal tail of Eck and then performing an *in vitro* kinase reaction on the immune complex. The phosphorylated protein was subjected to phosphoamino acid analysis, which confirmed that the majority of the phosphate was on tyrosine.

### *cek5*

*cek5* (*c*hicken *e*mbryo *k*inase) was isolated from a 10 day chicken embryo cDNA expression library probed with anti-phosphotyrosine antibodies (Pasquale, 1991). Antibodies to the Cek5 protein were raised against a β-gal fusion protein consisting of 759 amino acid residues (including all of the intracellular domain) and a synthetic peptide consisting of the ten amino acids from the C-terminal tail. Using these antibodies the Cek5 protein was found to have an apparent molecular mass of 120 kDa and its pattern of expression in the 10 day chicken embryo, determined by Western blotting, was found to be highest in the brain, marginally lower in the kidney, lung, thigh, gizzard and intestine, and lower still in the liver, heart and lens (Table I). In the adult chicken protein expression was found to be most abundant in the brain and detectable in most of the tissues seen in the embryo, but at a lower level. A more detailed study on the embryonic and newly hatched chicken brain revealed that expression decreases gradually during embryonic development and after hatching. Immunocytochemical staining showed that the Cek5 protein is expressed in regions that are rich in nerve cell processes especially in the hippocampus and the cerebellum (Pasquale *et al.*, 1992). Cek5 is specifically expressed in neurons and may play a role in neuronal maintenance in the chicken brain.

A variant of *cek5* was isolated from the same 10 day chicken embryo cDNA library and is termed $cek5^+$ (Sajjadi & Pasquale, 1993). This partial length cDNA variant codes for an *eph*-like receptor with an insert of 16 amino acids in the juxtamembrane region, which may be the result of alternative splicing. A Northern blot of 10-day-old chicken embryo brain and body tissue was screened with a probe specific for $cek5^+$ and one that would recognise both *cek5* and $cek5^+$. Using the probe that recognises both *cek5*s a 4.4 kb transcript was detected in 10 day embryonic brain and body tissues, with a 10 kb transcript also being detected in the brain. The $cek5^+$ probe detected the 4.4 kb transcript only, and this was expressed exclusively in the CNS. $cek5^+$ therefore appears to be a neuronal-specific variant of *cek5*.

### *sek*

Another *eph* family member, *sek* (*s*egmentally *e*xpressed *k*inase), seems to be involved in the development of the mouse hindbrain. *sek* was isolated from an 8.5 day mouse embryo cDNA library and the gene has been mapped to mouse chromosome 1 and human chromosome 2 (Gilardi-Hebenstreit *et al.*, 1992). Murine *sek* mRNA is 7.0 kb in size and was found to be most highly expressed in the adult mouse brain. However it was also detectable in the heart and lung, with a lower level of expression being seen in the kidney (Table I). A detailed study of the expression of mRNA in the developing mouse brain revealed *sek* is expressed initially in the forebrain and hindbrain but not in the midbrain, with expression becoming more restricted within the developing forebrain (Nieto *et al.*, 1992). *sek* also appears to be expressed in the developing neural tube of the spinal cord and *sek* may therefore have a role to play in the initial steps of neuronal differentiation in the spinal cord of the mouse. Later on in development *sek* may play a role in neuronal maintenance as is suggested for *cek5*.

### *cek4/mek4/hek*

Chicken *cek4* (isolated at the same time as *cek5*) encodes a 7.5 kb mRNA which was detectable in brain, head structures and body tissues of an 8 day chicken embryo (Sajjadi *et al.*, 1991). Expression of the 7.5 kb transcript was most pronounced in adult brain and retina, but was detectable in all other adult tissue except the liver (Sajjadi & Pasquale, 1993). *cek4* was used to isolate the mouse homologue termed *mek4* (*m*ouse *e*mbryo *k*inase) (Sajjadi *et al.*, 1991), not to be confused with *M*AP kinase/*E*RK *k*inase (MEK), which is responsible for phosphorylating the *e*xtracellular signal-regulated *k*inases (ERK) (Crews *et al.*, 1992). A cDNA coding for a soluble form of *mek4* was isolated at the same time as the usual membrane-spanning form. The soluble form consists of the extracellular domain only and possesses no transmembrane coding region. The *mek4* gene that codes for the full-length and secreted form of the receptor possesses an internal exon which encodes a polyadenylation signal. Use of this exon would result in the secreted form of *mek4* being transcribed. This phenomenon has been noted for various RTKs, including the EGF receptor, c-*erb*B-2 and some of the FGF receptors. There is evidence to suggest that expression of truncated receptor tyrosine kinases are developmentally regulated (Vu *et al.*, 1989), however the function of these secreted extracellular domains has not been determined. One suggestion is that they may help regulate the levels of growth factors surrounding the cell or alternatively they could bind to the full-length receptor and inhibit activation by preventing productive dimerisation (Petch *et al.*, 1990).

The *mek4* mRNA is 6.0 kb in length and expression is similar to *elk* in that the highest level is seen in the brain and a lower level is detected in the testis, but the mRNA found here is only 3.4 kb in length and may represent a third form of this receptor, which may again be the result of alternative splicing (Table I). No mRNA of the soluble form of *mek*4 was detected, and it may be that this form is expressed in a tissue-specific and/or a stage-specific manner. Further studies are required to confirm this.

The human homologue of *cek*4/*mek*4 is termed *hek*. This was cloned from a cDNA library prepared from mRNA obtained from a human pre-B-cell line LK63/C20$^+$ (a variant of the parental cell line, LK63) (Wicks *et al.*, 1992). A monoclonal antibody, III.A4, which recognises the human Hek protein, was made by immunising Balb/c mice with the LK63 cell line. This was then used to perform biochemical analysis on the Hek protein. Immunoprecipitation of labelled Hek from LK63 cells showed the mature protein to have a molecular mass of 135 kDa, and 95 kDa when deglycosylated. When Hek was immunoprecipitated from LK63 cells labelled *in vivo* with $^{32}$P, a weak band of 135 kDa was detected, suggesting that Hek had been phosphorylated to a low level. However, in attempts to find a specific ligand, no increase in phosphorylation of Hek was observed when cells were treated with a variety of cytokines (Boyd *et al.*, 1992).

Two approaches were taken to determine the distribution of the Hek protein in normal and tumour tissue. The first was using immunocytochemistry on frozen sections of solid human biopsy tissues and the second was immunofluorescence followed by flow cytometry on single-cell suspensions of haemopoietic cells and solid human tissues. The results showed that normal tissue (spleen, lymph node, bone marrow, tonsil, breast and brain) and some acute lymphoblastic leukaemia, breast, cervical, prostate, ovarian and renal carcinomas were negative for Hek protein expression, whereas 1/28 chronic lymphocytic leukaemias and 2/39 acute myeloid leukaemias were positive. These data suggest that Hek may play a role in some human haematopoietic cell tumours.

Northern blots of mRNA from LK63 and the T-cell line JM probed with the *hek* cDNA revealed a band of 5.5–6.0 kb. When Southern blot analysis was performed on DNA prepared from LK63 and LK63/C20$^+$, which express higher levels of *hek* than LK63 cells, no amplification or rearrangement of the *hek* gene was detected. Further studies should be undertaken to determine whether the *hek* gene is overexpressed and/or amplified in human haematopoetic cell tumours and/or solid human tumours.

### *hek*2

The *hek*2 gene was isolated using PCR technology. Human cDNAs from embryonic tissue were used as templates and the primers were designed to specifically recognise *eph*-like receptors. The predicted amino acid sequence on the *hek*2 gene is most similar to the partially sequenced *eph*-like receptor, *cek*10 (Figure 1), and the gene has been located to the distal end of human chromosome 3. Northern blot analysis of human tissue, using the *hek*2 probe, recognised a transcript of 4.6 kb. Expression was highest in pancreas, lung, placenta, brain and kidney, with lower expression being noted in heart, skeletal muscle and liver (Table I). *hek*2 transcripts were also detected in tumour cell lines of squamous and breast origin but not from epithelial cells of the lung or HeLa cells. A *hek*2 transcript was detected in A431 cells and lysate from these cells was used in an *in vitro* kinase assay using polyclonal antibodies which were raised against a synthetic peptide to the C-terminal end of the predicted Hek2 protein sequence. The phosphorylated Hek2 protein was determined to be approximately 130 kDa (Bohme *et al.*, 1993).

### Partially sequenced *eph* family members

Many partial cDNA sequences of putative receptors belonging to the *eph* family have been reported. *eek* (eph-and elk-related kinase) was isolated from a rat brain cDNA library and was used to isolate human *erk* (*e*lk-*r*elated *k*inase) (Chan & Watt, 1991). This should not be confused with the ERK proteins, which are *e*xtracellular signal-*r*egulated *k*inases which become phosphorylated by MEK (Crews *et al.*, 1992). *eek* mRNA was only detectable in the rat brain, whereas *erk* mRNA was highest in lung and lower in rat placenta, brain and kidney. Recently a longer clone of *erk* was isolated from a human gastric cancer cDNA library and found to differ from the original clone in one predicted amino acid residue (Iwase *et al.*, 1993). Northern blots of RNA prepared from the stomach of embryonic and adult rats plus mRNA from three cases of human gastric cancer were probed with this longer *erk* clone. It was found that *erk* was preferentially expressed in 16 day rat embryo stomach and weakly, if at all, in the adult forestomach and glandular stomach. *erk* expression was much higher in 3/3 human gastric cancers examined when compared with normal gastric tissue. *erk* may therefore play a role in human gastric cancer.

In another study using PCR technology five partial sequences coding for *eph*-like receptors, *tyro*1, 4, 5, 6 and 11, were identified using rat cDNA as the template (Lai & Lemke, 1991). When their predicted amino acid sequences are compared with all other Eph-like receptors it is noted that 100% identity exists between rat Tyro1 and mouse Sek/chicken Cek8 (see below). One hundred per cent identity is also shared between Tyro4 and human Hek, Tyro5 and human Erk and Tyro6 and human Hek2. Tyro11 is most closely related (93% identical) at the predicted amino acid level to human Hek2. These partial clones may therefore represent rat homologues of the given full-length Eph-like receptors. The expression of the various *tyro* mRNAs in adult and neonatal rat tissues was examined. *tyro*1 and 4 are preferentially expressed in the cells of the CNS and the level is fairly constant from embryo day 12 to adulthood for *tyro*1, whereas *tyro*4 expression drops sharply at birth. *tyro*5 mRNA is found exclusively in the neural tissue, and the level of expression falls shortly after birth. *tyro*6 mRNA is found in the brain, where expression is maximal at embryonic day 12, after which it gradually falls and by 10 days after birth is fairly constant. *tyro*11 has a different expression pattern and is found predominantly in the heart and kidney, with a lower level being detectable in neural tissue. Five partial length *eph*-like receptor cDNAs were isolated from the 10 day chicken embryo cDNA library, used to isolate *cek*4 and 5, and a 13 day chicken embryo brain cDNA library (Sajjadi & Pasquale, 1993). These have been termed *cek*6, *cek*7, *cek*8, *cek*9 and *cek*10. *cek*6 is thought to be the avian homologue of rat *elk*, while *cek*8 is considered to be the avian homologue of rat *tyro*1 and murine *sek*. *cek*10 is thought to be the avian homologue of human *hek*2, whereas *cek*7 and *cek*9 appear to be new *eph*-like receptors (see the phylogenetic tree in Figure 1). *cek*7 mRNAs were found to be mainly expressed in embryonic and adult brain. The highest level of *cek*8 mRNA expression is found in the adult chicken brain and retina. *cek*9 mRNA levels were highest in the adult chicken thymus, and lower in the brain, retina, kidney, lung and heart (Table I).

A variant form of *cek*10, termed *cek*10$^+$, was isolated. This variant possesses an insert of 15 amino acids in the juxtamembrane domain, similar to that seen with the *cek*5 variant, *cek*5$^+$. This variation may be the result of alternative splicing, but the significance of this is as yet unclear.

A partial *eph*-like receptor sequence was isolated using PCR technology. mRNA from a human breast cancer cell line was used as the template and the primers were degenerate oligonucleotides to protein kinases. One out of 32 tyrosine kinase-coding PCR products was found to belong to the *eph* family and was termed *tk*2 (Cance *et al.*, 1993). At the predicted amino acid level, 91% identity is shared between Tk2 and human Eck. The level of *tk*2 expression was beyond the limits of detection of a Northern blot of RNA prepared from various epithelial cell lines. However, *tk*2 expression could be detected in some of these cell lines when PCR technology was used, but when nine human primary and metastatic breast cancers were examined using this technique *tk*2 was undetectable.

### Conclusions

Owing to modern molecular biology techniques the *eph* family is rapidly expanding, and is presently the largest known family of RTKs. However, at present only very limited information as to their possible functions is available. It appears that *sek*, *cek*5, *elk*, *eek* and possibly *tyro*1, 4, 5 and 6 may have roles to play in the development of the brain and CNS, and in the maintenance of these tissues. As to the functions of the remaining family members, further studies must be undertaken before any conclusions can be made. Although there is preliminary evidence to suggest that *eph*, *hek*, *erk* and *elk* may be involved in some forms of human cancers, larger studies are necessary to confirm this. Other family members should also be investigated for mutations, gene amplification and/or overexpression in human carcinomas.

There is evidence to suggest that *cek*5, *cek*10 and *mek*4 may exist as alternatively spliced variants. The significance of the resulting truncated receptor, in the case of *mek*4, or receptors possessing inserts in the juxtamembrane region, as is seen with *cek*5$^+$ and *cek*10$^+$, is as yet unknown, however this warrants further investigation. The other *eph*-like receptors should also be examined for splice variants.

Once the ligands for the Eph-like receptors have been identified, biochemical studies will be possible and the true functions of this large family of 'putative' growth factor receptors will begin to be unravelled.

We wish to thank Alex Whittaker in the Biomedical Informatics Unit, ICRF, Lincoln's Inn Fields, London, for constructing the Eph family phylogenetic tree.

### References

BOHME, B., HOLTRICH, U., WOLF, G., LUZIUS, H., GRZESCHIK, K.H., STREBHARDT, K. & RUBSAMEN-WAIGMANN, H. (1993). PCR mediated detection of a new human receptor-tyrosine-kinase, HEK 2. *Oncogene*, **8**, 2857–2862.

BOYD, A.W., WARD, L.D., WICKS, I.P., SIMPSON, R.J., SALVARIS, E., WILKS, A., WELCH, K., LOUDOVARIS, M., ROCKMAN, S. & BUSMANIS, I. (1992). Isolation and characterisation of a novel receptor-type protein tyrosine kinase (*hek*) from a human pre-B cell line. *J. Biol. Chem.*, **267**, 3262–3267.

CANCE, W.G., CRAVEN, R.J., WEINER, T.M. & LIU, E.T. (1993). Novel protein kinases expressed in human breast cancer. *Int. J. Cancer.*, **54**, 571–577.

CHAN, J. & WATT, V.M. (1991). *eek* and *erk*, new members of the *eph* subclass of receptor protein tyrosine kinases. *Oncogene*, **6**, 1057–1061.

CREWS, C.M., ALESSANDRINI, A. & ERIKSON, R.L. (1992). The primary structure of *MEK*, a protein kinase that phosphorylates the ERK gene product. *Science*, **258**, 478–480.

DE SOETE, G. (1983). A least squares algorithm for fitting additive trees to proximity data. *Psychometrika*, **48**, 621–626.

DE SOETE, G. (1984). Additive tree representations of incomplete dissimilarity data. *Qual. Quant.*, **18**, 387–393.

GILARDI-HEBENSTREIT, P., NIETO, M.A., FRAIN, M., MATTEI, M.G., CHESTIER, A., WILKINSON, D.G. & CHARNAY, P. (1992). An *eph*-related receptor protein tyrosine kinase gene segmentally expressed in the developing mouse hindbrain. *Oncogene*, **7**, 2499–2506.

HANKS, S.K., QUINN, A.M. & HUNTER, T. (1988). The protein kinase family: conserved features and deduced phylogeny of the catalytic domains. *Science*, **241**, 42–52.

HIRAI, H., MARU, Y., HAGIWARA, K., NISHIDA, J. & TAKAKU, F. (1987). A novel putative tyrosine kinase receptor encoded by the *eph* gene. *Science*, **238**, 1717–1720.

IWASE, T., TANAKA, M., SUZUKI, M., NAITO, Y., SUGIMURA, H. & KINO, I. (1993). Identification of protein-tyrosine kinase genes preferentially expressed in embryo stomach and gastric cancer. *Biochem. Biophys. Res. Commun.*, **194**, 698–705.

LAI, C. & LEMKE, G. (1991). An extended family of protein tyrosine kinase genes differentially expressed in the vertebrate nervous system. *Neuron*, **6**, 691–704.

LEHTOLA, L., PARTANEN, J., SISTONEN, L., KORHONEN, J., WARRI, A., HARKONEN, P., CLARKE, R. & ALITALO, K. (1992). Analysis of tyrosine kinase mRNAs including four FGF receptor mRNAs expressed in MCF-7 breast-cancer cells. *Int. J. Cancer*, **50**, 598–603.

LEMOINE, N.R., BARNES, D.M., HOLLYWOOD, D.P., HUGHES, C.M., SMITH, P., DUBLIN, E., PRIGENT, S.A., GULLICK, W.J. & HURST, H.C. (1992). Expression of the *ERBB3* gene product in breast cancer. *Br. J. Cancer*, **66**, 1116–1121.

LETWIN, K., YEE, S.P. & PAWSON, T. (1988). Novel protein tyrosine kinase cDNAs related to *fps/fes* and *eph* cloned using anti phosphotyrosine antibody. *Oncogene*, **3**, 621–627.

LHOTAK, V., GREER, P., LETWIN, K. & PAWSON, T. (1991). Characterization of *elk*, a brain-specific receptor tyrosine kinase. *Mol. Cell. Biol.*, **11**, 2496–2502.

LINDBERG, R.A. & HUNTER, T. (1990). cDNA cloning and characterization of eck, an epithelial cell receptor protein-tyrosine kinase in the *eph/elk* family of protein kinases. *Mol. Cell Biol.*, **10**, 6316–6324.

MARU, Y., HIRAI, H., YOSHIDA, M.C. & TAKAKU, F. (1988). Evolution, expression, and chromosomal location of a novel receptor tyrosine kinase gene. *eph. Mol. Cell Biol.*, **8**, 3770–3776.

MARU, Y., HIRAI, H. & TAKAKU, F. (1990). Overexpression confers an oncogenic potential upon the *eph* gene. *Oncogene*, **5**, 445–447.

NIETO, M.A., GILARDI-HEBENSTREIT, P., CHARNAY, P. & WILKINSON, D.G. (1992). A receptor protein tyrosine kinase implicated in the segmental patterning of the hindbrain and mesoderm. *Development*, **116**, 1137–1150.

PASQUALE, E.B. (1991). Identification of chicken embryo kinase 5, a developmentally regulated receptor-type tyrosine kinase of the *eph* family. *Cell Regul.*, **2**, 523–534.

PASQUALE, E.B., DEERINCK, T.J., SINGER, S.J. & ELLISMAN, M.H. (1992). *cek*5, a membrane receptor-type tyrosine kinase, is in neurons of the embryonic and postnatal avian brain. *J. Neurosci.*, **12**, 3956–3967.

PAWSON, T. & BERNSTEIN, A. (1990). Receptor tyrosine kinases: genetic evidence for their role in *Drosophila* and mouse development. *Trends Genet.*, **6**, 350–356.

PETCH, L.A., HARRIS, J., RAYMOND, V.W., BLASBAND, A., LEE, D.C. & EARP, H.S. (1990). A truncated, secreted form of the epidermal growth factor receptor is encoded by an alternatively spliced transcript in normal rat tissue. *Mol. Cell Biol.*, **10**, 2973–2982.

PLOWMAN, G.D., CULOUSCOU, J.M., WHITNEY, G.S., GREEN, J.M., CARLTON, G.W., FOY, L., NEUBAUER, M.G. & SHOYAB, M. (1993). Ligand-specific activation of HER4/p180$^{erbB4}$, a fourth member of the epidermal growth factor receptor family. *Proc. Natl Acad. Sci. USA*, **90**, 1746–1750.

PRIGENT, S.A. & LEMOINE, N.R. (1992). The type 1 (EGFR-related) family of growth factor receptors and their ligands. *Prog. Growth Factor Res.*, **4**, 1–24.

SAJJADI, F.G., PASQUALE, E.B. & SUBRAMANI, S. (1991). Identification of a new *eph*-related receptor tyrosine kinase gene from mouse and chicken that is developmentally regulated and encodes at least two forms of the receptor. *New Biol.*, **8**, 769–778.

SAJJADI, F.G. & PASQUALE, E.B. (1993). Five novel avian *eph*-related tyrosine kinases are differentially expressed. *Oncogene*, **8**, 1807–1813.

ULLRICH, A. & SCHLESSINGER, J. (1990). Signal transduction by receptors with tyrosine kinase activity. *Cell*, **61**, 202–212.

VU, T.H., MARTIN, G.R., LEE, P., MARK, D., WANG, A. & WILLIAMS, L.T. (1989). Developmentally regulated use of alternative promoters creates a novel platelet-derived growth factor receptor transcript in mouse teratocarcinoma and embryonic stem cells. *Mol. Cell Biol.*, **9**, 4563–4567.

WICKS, I.P., WILKINSON, D., SALVARIS, E. & BOYD, A.W. (1992). Molecular cloning of *HEK*, the gene encoding a receptor tyrosine kinase expressed by human lymphoid tumor cell lines. *Proc. Natl Acad. Sci. USA*, **89**, 1611–1615.

YARDEN, Y. & ULLRICH, A. (1988). Growth factor receptor tyrosine kinases. *Annu. Rev. Biochem.*, **57**, 443–478.